# DNA Barcode Authentication of Devil’s Claw Herbal Dietary Supplements

**DOI:** 10.3390/plants10102005

**Published:** 2021-09-24

**Authors:** Genelle L. Diaz-Silveira, Joan Deutsch, Damon P. Little

**Affiliations:** Lewis B. and Dorothy Cullman Program for Molecular Systematics, The New York Botanical Garden, Bronx, NY 10458-5126, USA; nelleds@gmail.com (G.L.D.-S.); joandeutsch@optonline.net (J.D.)

**Keywords:** *Harpagophytum procumbens*, *Harpagophytum zeyheri*, mini-barcode, Pedaliaceae, *psbA-trnH*

## Abstract

Devil’s claw is the vernacular name for a genus of medicinal plants that occur in the Kalahari Desert and Namibia Steppes. The genus comprises two distinct species: *Harpagophytum procumbens* and *H. zeyheri*. Although the European pharmacopeia considers the species interchangeable, recent studies have demonstrated that *H. procumbens* and *H. zeyheri* are chemically distinct and should not be treated as the same species. Further, the sale of *H. zeyheri* as an herbal supplement is not legal in the United States. Four markers were tested for their ability to distinguish *H. procumbens* from *H. zeyheri*: *rbcL*, *matK*, nrITS2, and *psbA-trnH*. Of these, only *psbA-trnH* was successful. A novel DNA mini-barcode assay that produces a 178-base amplicon in *Harpagophytum* (specificity = 1.00 [95% confidence interval = 0.80–1.00]; sensitivity = 1.00 [95% confidence interval = 0.75–1.00]) was used to estimate mislabeling frequency in a sample of 23 devil’s claw supplements purchased in the United States. PCR amplification failed in 13% of cases. Among the 20 fully-analyzable supplements: *H. procumbens* was not detected in 75%; 25% contained both *H. procumbens* and *H. zeyheri*; none contained only *H. procumbens*. We recommend this novel mini-barcode region as a standard method of quality control in the manufacture of devil’s claw supplements.

## 1. Introduction

*Harpagophytum* (Pedaliaceae) is a genus of tuberous plants from the Kalahari Desert and Namibia Steppes that is commonly known as devil’s claw due to its hooked fruits [1]. The genus comprises two distinct species—*H. procumbens* and *H. zeyheri*—that have been separated on the basis of morphology [2,3,4] and chemistry [5]. *Harpagophytum* procumbens consists of two subspecies [3], *H. procumbens* subsp. *procumbens*, which occurs across Namibia, Botswana, and Northern South Africa, and *H. procumbens* subsp. *transvaalense*, which occurs only in the Limpopo region of South Africa. *Harpagophytum zeyheri* comprises three subspecies [3], *H. zeyheri* subsp. *zeyheri*, which is restricted in distribution to northeastern South Africa, and *H. zeyheri* subsp. *schijffii* and *H. zeyheri* subsp. *sublobatum*, which are both widely distributed across regions of Angola, Zambia, and Zimbabwe and the northern regions of Namibia and Botswana.

There are unsubstantiated reports of possible hybridization in the few places where *H. procumbens* and *H. zeyheri* are sympatric [4,6,7]. Although purporting to demonstrate hybridization, RAPD and ISSR data [7] are, at best, inconclusive: no species-specific genotype groups were detected [7], thus a definitive pattern of hybridization cannot possibly be observed; the published Principal Component Analysis [7]—which is inappropriate for detecting hybridization [8,9]—identifies five putative hybrids, but only one individual is truly intermediate while several non-hybrid samples are equally or more intermediate than the putative hybrids; and the published UPGMA dendrogram [7] refutes the hypothesis of hybridization because it nests the putative hybrids well within the two parental clusters rather than at the cluster base where hybrids are expected to appear [10].

In addition, morphological data [4] purportedly demonstrate hybridization, but they are not statistically significant: the published Discriminant Function Analysis (DFA) [4] improperly implemented DFA such that hybrids were assumed to be present rather than using DFA to test that supposition. In addition, measurements that violate the Gaussian distribution assumed by DFA [11] were included. If DFA is conducted on the five characteristics that do not deviate [12] significantly (*p* > 0.01) from the Gaussian distribution (arm width, seed column height, fruit length, fruit width, and fruit circumference), the putative hybrids [4] are classified without evidence of intermediacy (*pp* ≥ 0.99999). Independent of the improperly implemented DFA, no statistical test was conducted to determine if the putative hybrids were truly intermediate [4]: the character count procedure [9] employing the sign [13] and Scheffé [14] tests (*p* = 0.05) does not indicate intermediacy for any characters and thus no trace of hybridity was detected (*p* = 1.0).

Given this critical review, there are no published data showing evidence of hybridization between *Harpagophytum* species and further study of additional specimens and characteristics is needed to determine if hybridization does indeed occur.

Devil’s claw has traditionally been used to treat dyspepsia, fever, constipation, hypertension, and venereal disease [1]. Commercial preparations of *H. procumbens* are sold to treat arthritis in both the European and United States markets [15]. *Harpagophytum zeyheri* cannot be legally sold as an herbal supplement in the United States [16] but it was appended to the European Pharmacopeia [17]. Both species are wild sourced—primarily from Namibia [18]. 

Although clinical trials have demonstrated the efficacy of *H. procumbens* for musculoskeletal pain relief [19,20,21,22], animal and in vitro studies have produced conflicting results [23,24,25,26,27]. The suspected active compounds—harpagoside, harpagide, 8-p-coumaroyl-harpagide, and acteoside—inhibit cyclooxygenase (*COX*) 1 and 2 [28,29,30] and the pro-inflammatory cytokines tumor necrosis factor-α (*TNF-α*), interleukin-Iβ (*IL-Iβ*), and interleukin-6 (*IL-6*) [31,32]. Harpagoside is the main anti-inflammatory agent, but it is less effective in isolation [31] and thus the constituents of *H. procumbens* are thought to have synergistic effects [33]. 

Commercial herbal supplements are most frequently sold as dry fragments or powders. As a result, the authentication of these materials has traditionally relied upon macro- and microscopic morphological examination along with chemical assays for specific compounds or classes of compounds [34]. In the last two decades, DNA-based assays have become more common with assays for specific plants (e.g., molecular marker-based methods that utilize simple sequence repeats (SSR) or single nucleotide polymorphisms (SNP)) and general untargeted analysis techniques (e.g., short fragment sequencing methods such as whole metagenome analysis and metabarcoding) now being prominently used [35,36,37]. DNA barcoding has emerged as a preferred method of herbal supplement authentication due to the fact that it generally works well with highly fragmented DNA from high-copy regions (e.g., plastid), can detect multiple species at once, and is relatively inexpensive. These characteristics make the method ideal for assaying the DNA in highly degraded herbal products.

Devil’s claw supplements are sold mainly in capsule or tablet form [38]. Thus, it is impossible to determine which species they contain without additional analysis. A reliable identification method to ensure correct labeling is needed. We aim to create and test a DNA mini-barcode assay for both *Harpagophytum* species.

## 2. Results

### 2.1. Reference Sequences

Reference sequences from four markers were generated from 39 morphologically identifiable specimens (Table 1). In total, 17 *rbcL*, 23 *matK*, 22 nrITS2, and 35 *psbA-trnH* barcodes were produced. Median sequence quality (B*_30_* [39]) exceeds the requirements of the BARCODE data standard (version 2.3 [40]): 0.841 (IQR 0.682–0.936) for *rbcL*, 0.891 (IQR 0.649–0.941) for *matK*, 0.849 (IQR 0.682–0.879) for nrITS2, and 0.845 (IQR = 0.466–0.890) for *psbA-trnH*.

**Table 1:** Morphologically identifiable reference samples used to generate *rbcL*, *matK*, nrITS2, *psbA-trnH* and/or *psbA-trnH* mini-barcode sequences and to validate the *psbA-trnH* mini-barcode. Standard herbarium codes are used [41]. Cultivated specimens are indicated, all others are presumed to be wild collected. All sequences except EU531713 [42] were produced for this study.

Species	Voucher Specimen	Locality	Sample Type	GenBank Accession			
				*rbcL*	*matK*	nrITS2	*psbA-trnH*
*Dicerocaryum zanguebarium*	Loeb and Koch 339 (NY)	Namibia: Oshikango	reference and validation	—	—	—	KT717163
*Harpagophytum procumbens*	Allen 308 (M0)	Botswana: Orapa	reference and validation	—	KT717103	KT717127	KT717148
*Harpagophytum procumbens*	Davidse and Loxton 6296 (MO)	Namibia: Keetmanshoop	reference and validation	KT717178	KT717109	KT717133	KT717153
*Harpagophytum procumbens*	de Koning 8142 (MO)	Mozambique: Chigubo	reference	—	KT717110	—	—
*Harpagophytum procumbens*	Dinter 396 (MO)	Namibia: Okahandja	reference and validation	—	—	—	KT717150
*Harpagophytum procumbens*	Grignon 239 (MO)	Botswana: Ghanzi	reference and validation	KT717174	KT717104	KT717128	KT717149
*Harpagophytum procumbens*	Hardy 6575 (MO)	Namibia: Aranos	reference and validation	KT717168	KT717095	KT717124	KT717154
*Harpagophytum procumbens*	Herman 1264 (MO)	South Africa: Blouberg Privaatnatuurreserwe	reference and validation	KT717176	KT717107	KT717131	KT717151
*Harpagophytum procumbens*	Lavranos and Bleck 22701 (MO)	Namibia: Otjiwarongo	reference and validation	KT717177	KT717108	KT717132	KT717152
*Harpagophytum procumbens*	Lavranos and Bleck 22703 (MO)	Namibia: Khorixas	reference and validation	KT717173	KT717102	KT717126	KT717147
*Harpagophytum procumbens*	Leach 10682 (MO)	Zimbabwe: Beit Bridge	reference	—	KT717099	—	—
*Harpagophytum procumbens*	Long and Rae 44 (MO)	Botswana: Jwaneng	reference and validation	KT717171	KT717101	KT717120	KT717145
*Harpagophytum procumbens*	Ngoni 257 (MO)	Botswana: Mosu	reference	—	KT717105	KT717129	KY706349
*Harpagophytum procumbens*	Owens 19 (MO)	Botswana: Deception Valley	reference and validation	KT717172	KT717096	KT717125	KT717146
*Harpagophytum procumbens*	Rodin 3539 (NY)	South Africa: Vryburg	reference	—	—	—	KY706351
*Harpagophytum procumbens*	Rogers s.n. (MO)	South Africa: Bellville	reference	—	KT717097	—	KY706348
*Harpagophytum procumbens*	Sidey 305 (MO)	South Africa: Fauresmith	reference and validation	KT717169	KT717098	KT717119	KT717143
*Harpagophytum procumbens*	Skarpe S-319 (MO)	Botswana: Hukuntsi	reference and validation	KT717170	KT717100	KT717123	KT717144
*Harpagophytum procumbens*	Smuts and Gillelt 2130 (MO)	South Africa: Rooikop	validation	—	—	—	—
*Harpagophytum procumbens*	Venter 9637 (MO, NY)	South Africa: Glen Agricultural College	reference	KT717175	KT717106	KT717130	KY706350
*Harpagophytum zeyheri*	Germishuizen 00733 (MO)	South Africa: Bamboeskloof	reference and validation	—	KT717114	KT717122	KT717159
*Harpagophytum zeyheri*	Germishuizen 990 (MO)	South Africa: Vaalwater	reference and validation	KT717183	—	KT717138	KT717160
*Harpagophytum zeyheri*	Luwiika et al. 335 (MO)	Zambia: Lukona Basic School	reference	—	KT717116	KT717137	—
*Harpagophytum zeyheri*	Mashasha 111 (MO)	Zimbabwe: Victoria Falls	reference and validation	KT717179	KT717111	KT717134	KT717155
*Harpagophytum zeyheri*	Mogg 37171 (MO)	South Africa: Sandsloot	reference and validation	KT717182	KT717113	KT717136	KT717157
*Harpagophytum zeyheri*	Moyo 7 (MO)	Zimbabwe: Victoria Falls	reference	—	—	KT717118	—
*Harpagophytum zeyheri*	Norlindh and Weimarck 5234 (NY)	South Africa: Pietersburg	reference	—	—	—	KY706353
*Harpagophytum zeyheri*	Rodin 9140 (MO)	Namibia: Rundu	reference and validation	KT717184	KT717115	KT717121	KT717158
*Harpagophytum zeyheri*	Rushworth 110 (MO)	Zimbabwe: Dina Pan	reference and validation	KT717180	KT717094	KT717135	KT717156
*Harpagophytum zeyheri*	Yalala 300 (MO)	Botswana: Mahalapye	reference	KT717181	KT717112	KT717117	KY706352
*Josephinia euginiae*	Michell and Boyce 3144 (MO)	Australia: Nitmiluk National Park	reference and validation	—	—	—	KT717162
*Pedaliodiscus macrocarpus*	Luke et al. TPR 73 (MO)	Kenya: Tana River National Primate Reserve	reference and validation	—	—	—	KT717139
*Pedalium murex*	Comanor 608 (NY)	Sri Lanka: Potuvil—Panama Road	reference and validation	—	—	—	KT717140
*Pterodiscus auranthacus*	Seydel 4135 (NY)	Namibia: Windhoek	reference and validation	—	—	—	KT717141
*Pterodiscus speciosus*	Zietsman 4079 (NY)	South Africa: Hoopstad	reference and validation	—	—	—	KT717142
*Rogeria adenophylla*	Seydel 4368 (NY)	Namibia: Windhoek	reference and validation	—	—	—	KT717167
*Sesamum indicum*	Donmez 9932 (NY)	Turkey: Kula	reference and validation	—	—	—	KT717164
*Sesamum indicum*	Nesbitt 1939 (RNG)	—	reference	—	—	—	EU531713
*Sesamum radiatum*	Thomas 10563 (NY)	Brazil: Ilhéus	reference and validation	—	—	—	KT717165
*Sesamum triphyllum*	Zietsman and Peyper 4061 (NY)	South Africa: Petrusburg	reference and validation	—	—	—	KT717161
*Uncarina grandidieri*	Falk 97001 (NY)	cultivated	reference and validation	—	—	—	KT717166

**Figure 1:**
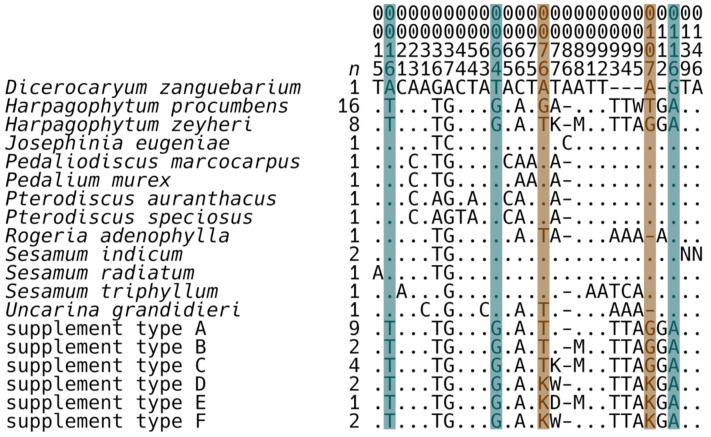
Variable nucleotides within the *psbA-trnH* mini-barcode (voucher information is in Table 1; herbal dietary supplement information is in Table 2; the full alignment is in Figure A1). Alignment positions are numbered vertically. Bases identical to the first sequence are indicated with “.”. Variable bases are indicated with standard International Union of Pure and Applied Chemistry (IUPAC) codes: D = {AGT}, K = {GT}, M = {AC}, N = {ACGT}, and W = {AT}. The number of sequences summarized (*n*) for each species/supplement type is indicated. Alignment positions that unambiguously distinguish *Harpagophytum* from all other Pedaliaceae (16, 64, and 116) are highlighted in blue. The alignment positions 76 and 107—which distinguish between the two *Harpagophytum* species—are highlighted in orange.

**Figure A1:**
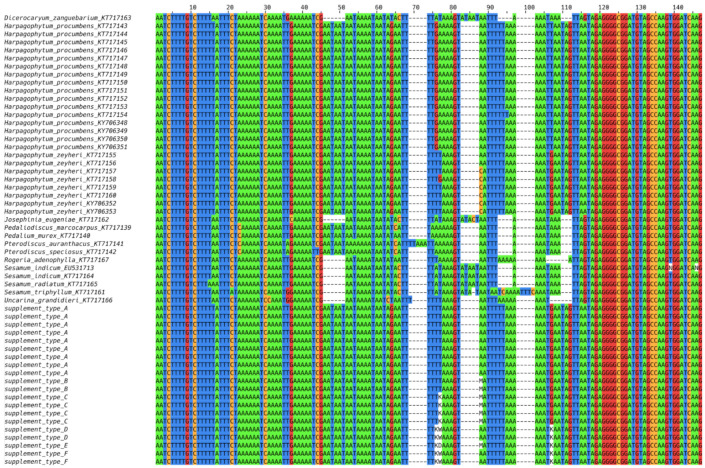
Full *psbA-trnH* mini-barcode alignment.

Within *Harpagophytum*, variation was only observed in *psbA-trnH* (Figure 1, Figure A1). *Harpagophytum* can be unambiguously distinguished from all other Pedaliaceae by alignment positions 16, 64, and 116. The two *Harpagophytum* species can be differentiated by alignment positions 76 and 107. Intraspecific variation was observed in reference samples of both *H. procumbens* (alignment position 95) and *H. zeyheri* (alignment positions 77 and 88). Only one of these variants is exactly correlated with geography or current taxonomy: position 77 distinguishes *H. zeyheri* subsp. *suboblatum* (sample from Namibia) from *H. zeyheri* subsp. *zeyheri* (samples from South Africa). No samples of *H. zeyheri* subsp. *schijffii* were available for examination.

Across Pedaliaceae, the *psbA-trnH* alignment is 427 columns and has 13 unique insertion/deletion (indel) events ranging from 1–13 bases (median 6; IQR 4–7). The unaligned sequences range from 367–394 bases (median 383; IQR 373–383). Within *Harpagophytum*, the *psbA-trnH* sequences are uniformly 383 bases without any evidence of indels.

### 2.2. Mini-Barcode Validation

Validation *psbA-trnH* mini-barcode (*n* = 30) median sequence quality was 0.569 (IQR 0.532–0.587). BRONX [43] was able to correctly identify all *H. procumbens* validation samples and exclude *H. procumbens* as a possible identification for all other validation samples (*n* = 13 *H. procumbens*; *n* = 17 other species; specificity = 1.00 [95% confidence interval = 0.80–1.00]; sensitivity = 1.00 [95% confidence interval = 0.75–1.00]; [44]). The absolute consistency of alignment positions 16, 64, 76, 107, and 116 prevent infraspecific variation from having any bearing on *Harpagophytum* species identification.

### 2.3. An Analysis of Herbal Supplements

Amplifiable DNA was extracted from 20 of 23 (87%) herbal supplements. Amplification success was significantly correlated with the reports of root extract on product labels (McNemar test [45]; *p* = 0.04331; Table 2). The failure rate for samples labeled as having root extract (17%) was nearly double that of samples without root extract (9%; Table 2).

**Table 2:** Herbal dietary supplement label ingredients and *psbA-trnH* mini-barcode determination. Supplement sequence type corresponds to those in Figure 1. If Latin names were not provided on the product label, the Latin name was determined using [16]. Despite being noted on some labels, the sale of supplements containing *H. zeyheri* is not legal in the United States.

Supplement Sequence Type	Label Species	Devil’s Claw Material Type	Contains *H. procumbens*	Contains *H. zeyheri*
A	*Harpagophytum procumbens, Curcuma longa, Crataegus oxyacantha, Arctium lappa, Smilax febrifuga, Yucca schidigera, Zingiber officinale,* and *Vaccinium myrtillus*	root extract	no	yes
A	*Harpagophytum procumbens*	root	no	yes
A	*Harpagophytum procumbens*	root	no	yes
A	*Boswellia serrata, Curcuma longa*, and *Harpagophytum procumbens*	root extract	no	yes
A	*Boswellia serrata, Uncaria tomentosa, Harpagophytum procumbens, Yucca schidigera, Gymnema sylvestre, Curcuma longa, Camellia sinensis*, and *Oryza sativa*	root	no	yes
A	*Harpagophytum procumbens* and *Oryza sativa*	root extract	no	yes
A	*Harpagophytum procumbens*	root extract	no	yes
A	*Harpagophytum procumbens*	root	no	yes
A	*Harpagophytum procumbens, Boswellia serrata, Curcuma longa*, and *Tanacetum parthenium*	root extract	no	yes
B	*Harpagophytum procumbens*	root	no	yes
B	*Harpagophytum procumbens*	root extract	no	yes
C	*Harpagophytum procumbens*	root	no	yes
C	*Harpagophytum procumbens* and *Oryza sativa*	root extract	no	yes
C	*Harpagophytum procumbens*	root	no	yes
C	*Harpagophytum procumbens*	root	no	yes
D	*Harpagophytum procumbens*	root	yes	yes
D	*Harpagophytum procumbens* and/or *Harpagophytum zeyheri*	root extract	yes	yes
E	*Harpagophytum procumbens*	root and root extract	yes	yes
F	*Harpagophytum procumbens*	root	yes	yes
F	*Harpagophytum procumbens* and/or *Harpagophytum zeyheri*	root extract	yes	yes
—	*Harpagophytum procumbens*	root	unknown	unknown
—	*Polygonum cuspidatum, Curcuma longa, Zingiber officinale, Camellia sinensis, Harpagophytum procumbens*, and *Salix alba*	root extract	unknown	unknown
—	*Harpagophytum procumbens*	root extract	unknown	unknown

PCR products were successfully sequenced for all 20 amplifiable supplements: mini-barcode median sequence quality was 0.561 (IQR 0.451–0.587)—very similar to the quality of the validation samples.

*Harpagophytum zeyheri* was found in all 20 fully-analyzable samples: all supplements contained either *H. zeyheri* (75%; 15/20; Types A, B, and C; a “T” at alignment position 76 and a “G” at alignment position 107; Figure 1, Table 2) or a combination of *H. procumbens* and *H. zeyheri* (25%; 5/20; Types D, E, and F; a “K” [“G” and “T”] at alignment positions 76 and 107; Figure 1, Table 2); no supplements contained only *H. procumbens*.

**Figure 2:**
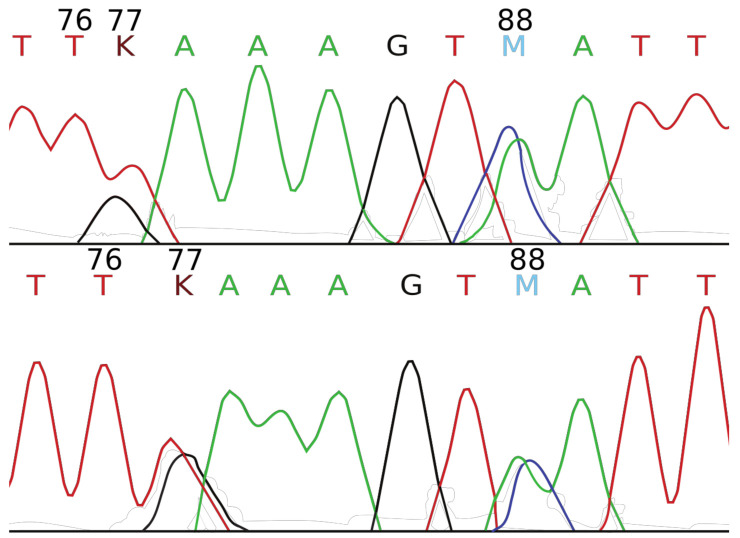
Portions of forward (top) and reverse (bottom) Sanger sequencing chromatograms demonstrating polymorphic positions (alignment positions 77 and 88) in herbal supplement mini-barcode sequences of a Type C sequence. Diagnostic nucleotides (Figure 1) are indicated by their alignment position; “A” = green; “G” = black; “K” = maroon {GT}; “M” = indigo {AC}; “T” = red. Despite the supplement being labeled as containing only *H. procumbens*, alignment position 76 indicates that this sample is composed exclusively of *H. zeyheri*.

Types A, B, and C contain *H. zeyheri* haplotypes that exhibit the same variation found in the reference samples. Type A is composed of samples that contain only one *H. zeyheri* haplotype, while types B and C are mixtures of *H. zeyheri* haplotypes (e.g., Figure 2). In contrast, types D, E, and F are mixtures of *H. procumbens* and *H. zeyheri* haplotypes. Type E contains one *H. procumbens* haplotype and two *H. zeyheri* haplotypes (a “D” [“A”, “G” and “T”] at alignment position 77; Figure 1).

## 3. Discussion

The *psbA-trnH* mini-barcode absolutely differentiates *Harpagophytum* from all other Pedaliaceae (Figure 1: blue highlighted positions 16, 64, and 116) and in turn *H. procumbens* and *H. zeyheri* from one another (Figure 1: orange highlighted positions 76 and 107). Thus, the species have consistent character state differences and can be considered distinct phylogenetic species [46]. The absolute consistency of *psbA-trnH* mini-barcode alignment positions 16, 64, 76, 107, and 116 prevent infraspecific variation from having any bearing on repeatable *Harpagophytum* species identification (specificity = 1.00 [95% confidence interval = 0.80–1.00]; sensitivity = 1.00 [95% confidence interval = 0.75–1.00]). Although there are reports of possible interbreeding between the two *Harpagophytum* species [4,6,7], the pattern observed here is inconsistent with hybridization because the morphological and molecular species identifications exactly match. No intermediate morphological phenotypes have been confirmed either in the literature or in our research, suggesting that hybrids, if they exist, have retained strong morphological similarity to one of the parental species. Therefore, absolute rejection of the hybridization hypothesis would require the investigation of multiple biparentally inherited molecular markers. Given the lack of support for the supposition of hybridization in the data, the regulatory distinction between *H. procumbens* and *H. zeyheri* in the United States [16] can be enforced.

The variation within the *psbA-trnH* mini-barcode used to differentiate between the two *Harpagophytum* species could be assayed using molecular techniques other than the Sanger sequencing method demonstrated here. For instance, one could use PCR-RFLP with *AseI* (5′-ATTAAT-3′) to assay alignment position 107 (*H. procumbens* will cut, but *H. zeyheri* will not); RT-PCR with specific primers and/or probes targeted to alignment positions 16, 64, 76, 107, and/or 116; or short read genome skimming (e.g., Illumina) with appropriate bioinformatic postprocessing to find alignment positions 16, 64, 76, 107, and 116 in the output sequences. Depending upon the needs of the user, each of these techniques could be conducted in such a way as to quantify the relative or absolute amounts of DNA from each species present in the sample.

The observed mini-barcode PCR amplification failure rate from herbal supplements of 13% is a bit high compared to the 3–10% reported for similar studies [47,48,49]. Although the processing of plant materials for herbal supplement manufacturing frequently results in DNA fragmentation and destruction [50,51,52,53,54,55,56,57,58,59,60,61,62,63,64,65,66,67,68,69,70] that can prevent amplification, the processing techniques used for devil’s claw may be more damaging than those used for other herbal supplements studied thus far—which is supported by the significant correlation between reports of root extract (a relatively damaging technique [70]) on product labels and PCR failure (McNemar test [45]; *p* = 0.04331; Table 2). It is also possible that some, or all, of the high rate of PCR failure can be attributed to the amount of recoverable DNA in devil’s claw tap roots being low and/or less enzymatically accessible in comparison to aerial parts as is the case in carrot (*Daucus carota*) tap roots [71,72].

Labels of only two of the 20 analyzable supplements (Table 2) list *Harpagophytum zeyheri*, but *H. zeyheri* was found in all 20 fully-analyzable samples. Somehow the two, predominantly allopatric [1], species were mixed. Although *H. zeyheri* can be legally sold in the European Union [17], it cannot be sold in the United States [16].

Bulk materials of devil’s claw are usually sold in a morphologically unidentifiable state [1,5]. Thus, a chemical test that measures the relative quantity of harpagoside and 8-p-coumaroyl-harpagide is often used to distinguish between bulk materials from the two species [73]. The data that purport to validate the assay were not analyzed statistically [73]. Unfortunately, the data do not statistically differentiate between the *Harpagophytum* species (Mann–Whitney test [74]; *p* = 0.1386)—perhaps due to the miniscule sample size (*n* = 5). Therefore, this chemical assay cannot be considered reliable. Revalidation with additional, morphologically identifiable and vouchered samples may redeem this assay for harpagoside and 8-p-coumaroyl-harpagide.

Due to the legal status of *H. zeyheri* in the United States, it is imperative that supplement manufacturers employ a robust method of quality control to evaluate all devil’s claw supplements sold. Because the mini-barcode presented here is reliable, cost-efficient, and simple to use, we recommend it as a standard method of quality control instead of the relative quantity of harpagoside and 8-p-coumaroyl-harpagide.

## 4. Materials and Methods

A barcode reference database of *rbcL*, *matK*, nrITS2, and *psbA-trnH* sequences was created from morphologically identifiable samples of Pedaliaceae. Specimen identifications followed standard references [3,6,75]. Sequences outside *Harpagophytum* were sampled from close (*Pterodiscus*, *Pedaliodiscus*, *Pedalium*, *Uncarina*, and *Rogeria*) and distant relatives (*Dicerocaryum*, *Josephinia*, and *Sesamum*; Table 1; [76]).

Validation samples were chosen arbitrarily (*n* = 30; Table 1). Herbal supplements (capsules and compression tablets) were purchased online.

A *psbA-trnH* mini-barcode was designed from all Pedaliaceae reference sequences. The mini-barcode is anchored within the intergenic spacer (alignment positions 1–122) and extends into *trnH* (alignment positions 123–147; Figure A1). This region was selected for its compactness and discriminatory power.

DNA was isolated [48] from leaves of reference and validation samples and powdered herbal supplements. Markers were amplified using the polymerase chain reaction (PCR). Each 15 µL reaction contained 1.5 µL PCR buffer (200 mM tris pH 8.8, 100 mM KCl, 100 mM (NH_4_)_2_SO_4_, 20 mM MgSO_4_, 1% (*v*/*v*) Triton X-100, 50% (*w*/*v*) sucrose, 0.25% (*w*/*v*) cresol red, and 0.25 µg/µL BSA), 0.2 mM of each dNTP, 1.0 µM of each amplification primer, 0.5 units of *Taq* polymerase, and 0.5 µL DNA. Primer sequences and cycling conditions are given in Table 3 and Table 4.

PCR products were treated with ExoSapIt (ThermoFisher, Waltham, MA), and sequenced bidirectionally on a 3730 automated sequencer (ThermoFisher) using the amplification primers and BigDye 3.1 (ThermoFisher).

KB 1.4 (ThermoFisher) was used to generate base calls and quantity values from raw chromatograms. Contigs were assembled and edited with Sequencher (version 5.2.3; Gene Codes, Ann Arbor, MI). Sequence quality was determined using *B* (version 1.2; [39]) with expected coverage (*x*) set to the number of reads. Newly generated mini-barcode sequences were compared to reference sequences using BRONX (version 2.0; [43]). R version 3.3.1 (http://www.R-project.org, accessed on 21 August 2021) was used to calculate discriminant function analysis [11], the Mann–Whitney test [74], the McNemar test [45], the Scheffé [14] test, the Shapiro–Wilk test [12], the sign test [13], and specificity and sensitivity [44].

## Figures and Tables

**Table 3:** PCR primers used for amplification and sequencing.

Marker	Primer Name	Sequence (5′–3′)	Source
*matK*	1R	ACCCAGTCCATCTGGAAATCTTGGTTC	K.J. Kim (pers. com.)
*matK*	3F	CGTACAGTACTTTTGTGTTTACGAG	K.J. Kim (pers. com.)
nrITS2	S2F	ATGCGATACTTGGTGTGAAT	[77]
nrITS2	S3R	GACGCTTCTCCAGACTACAAT	[77]
*psbA-trnH*	psbAF	GTTATGCATGAACGTAATGCTC	[78]
*psbA-trnH*	trnHR	CGCGCATGGTGGATTCACAAATC	[78]
*psbA-trnH* mini-barcode	F	GAAGATAAATGAAATGATTGAAATGC	novel
*psbA-trnH* mini-barcode	R	TGGATTCACAAATCCACTGC	novel
*rbcL*	32F	TTGGATTCAAAGCTGGTGTT	[79]
*rbcL*	a_F	ATGTCACCACAAACAGAGACTAAAGC	[80]
*rbcL*	ajf634R	GAAACGGTCTCTCCAACGCAT	[81]

**Table 4:** PCR cycling conditions used. Amplification reactions used an initial denaturation of 150 s at 95 °C and a final extension of 600 s at 72 °C (*psbA-trnH* used 64 °C). Primer names correspond to those in Table 3.

Marker	Primers	Cycling
*matK*	1R & 3F	10 × {30 s, 95 °C; 30 s, 56 °C; 30 s, 72 °C}; 25 × {30 s, 88 °C; 30 s, 56 °C; 30 s, 72 °C}
nrITS2	S2F & S3R	35 × {30 s, 95 °C; 30 s, 56 °C; 30 s, 72 °C}
*psbA-trnH*	psbAF & trnHR	10 × {30 s, 95 °C; 120 s, 55 °C}; 23 × {45 s, 90 °C; 120 s, 55 °C}
*psbA-trnH* mini-barcode	F & R	35 × {30 s, 95 °C; 120 s, 58 °C}
*rbcL*	32F & ajf634R	35 × {30 s, 95 °C; 30 s, 58 °C; 30 s, 72 °C}
*rbcL*	a_F & ajf634R	35 × {30 s, 95 °C; 30 s, 58 °C; 30 s, 72 °C}

## Data Availability

Sequences were deposited in GenBank accessions KT717094–KT717184 and KY706348–KY706353.

## References

[1] Stewart K.M., Cole D. (2005). The commercial harvest of Devil’s Claw (*Harpagophytum* spp.) in Southern Africa: The Devil’s in the details. J. Ethnopharmacol..

[2] Decaisne J. (1865). Revue du groupe des pedalinees. Ann. Sci. Nat. Bot..

[3] Ihlenfeldt H.-D., Hartmann H. (1970). Die gattung *Harpagophytum* (Burch.) DC. Ex Meissn. (Monographie Der Afrikanischen Pedaliaceae II). Mitt. Aus Dem Staatsinst. Fur Allg. Bot. Hambg..

[4] Muzila M., Setshogo M.P., Mpoloka S.W. (2011). Multivariate Analysis of *Harpagophytum* DC. Ex Meisn (Pedaliaceae) based on fruit characters. Int. J. Biodivers. Conserv..

[5] Mncwangi N.P., Viljoen A.M., Zhao J., Vermaak I., Chen W., Khan I. (2014). What the devil is in your phytomedicine? Exploring species substitution in *Harpagophytum* through chemometric modeling of 1H-NMR and UHPLC-MS datasets. Phytochemistry.

[6] Ihlenfeldt H.-D. (1988). Pedaliaceae. Flora Zambesiaca.

[7] Muzila M., Werlemark G., Ortiz R., Sehic J., Fatih M., Setshogo M., Mpoloka W., Nybom H. (2014). Assessment of diversity in *Harpagophytum* with RAPD and ISSR markers provides evidence of introgression. Hereditas.

[8] Pimental R.A. (1981). A comparative study of data and ordination techniques based on a hybrid swarm of sand verbenas (*Abronia* Juss.). Syst. Zool..

[9] Wilson P. (1992). On inferring hybridity from morphological intermediacy. Taxon.

[10] McDade L.A. (1997). Hybrids and phylogenetic systematics III. Comparison with distance methods. Syst. Bot..

[11] Fisher R.A. (1936). The use of multiple measurements in taxonomic problems. Ann. Eugen..

[12] Shapiro S.S., Wilk M.B. (1965). An analysis of variance test for normality (complete samples). Biometrika.

[13] Arbuthnott J. (1710). An argument for divine providence, taken from the constant regularity observ’d in the births of both sexes. Philos. Trans..

[14] Scheffé H. (1953). A method for judging all contrasts in the analysis of variance. Biometrika.

[15] Marshall N.T. (1998). Searching for a Cure: Conservation of Medicinal Wildlife Resources in East and Southern Africa.

[16] McGuffin M., Kartesz J.T., Leung A.Y., Tucker A.O. (2000). Herbs of Commerce.

[17] Grote K. (2003). The Increased Harvest and Trade of Devil’s Claw (Harpagophytum procumbens) and Its Impacts on the Peoples and Environment of Namibia, Botswana and South Africa.

[18] Raimondo D., Donaldson J. (2002). The Trade, Management and Biological Status of Harpagophytum Spp. in Southern African Range States.

[19] Chantre P., Cappelaere A., Leblan D., Guedon D., Vandermander J., Fournie B. (2000). Efficacy and tolerance of *Harpagophytum procumbens* versus diacerhein in treatment of osteoarthritis. Phytomedicine.

[20] Chrubasik S., Thanner J., Künzel O., Conradt C., Black A., Pollak S. (2002). Comparison of outcome measures during treatment with the proprietary *Harpagophytum* extract Doloteffin^®^ in patients with pain in the lower back, knee or hip. Phytomedicine.

[21] Frerick H., Schmidt U. (2001). Stufenschema bei coxarthrose. Der Kassenarzt.

[22] Wegener T., Lüpke N. (2003). Treatment of patients with arthrosis of hip or knee with an aqueous extract of Devil’s Claw (*Harpagophytum procumbens* DC). Phytother. Res..

[23] Andersen M.L., Santos E.H.R., Maria de Lourdes V.S., da Silva A.A.B., Tufik S. (2004). Evaluation of acute and chronic treatments with *Harpagophytum procumbens* on Freund’s adjuvant–induced arthritis in rats. J. Ethnopharmacol..

[24] Baghdikian B., Lanhers M.C., Fleurentin J., Ollivier E., Maillard C., Balansard G., Mortier F. (2007). An analytical study and anti–inflammatory and analgesic effects of *Harpagophytum procumbens* and *Harpagophytum zeyheri*. Planta Med..

[25] Kundu J.K., Mossanda K.S., Na H.-K., Surh Y.-J. (2005). Inhibitory effects of the extracts of *Sutherlandia frutescens* (L.) R. Br. and *Harpagophytum procumbens* DC. on phorbol ester–Induced *COX-2* expression in mouse skin: *AP-1* and *CREB* as potential upstream targets. Cancer Lett..

[26] McLeod D.W., Revell P., Robinson B.V. (1979). Investigations of *Harpagophytum procumbens* (Devil’s Claw) in the treatment of experimental inflammation and arthritis in the rat. Br. J. Pharmacol..

[27] Whitehouse L.W., Znamirowska M., Paul C.J. (1983). Devil’s Claw (*Harpagophytum procumbens*): No evidence for anti–inflammatory activity in the treatment of arthritic disease. Can. Med Assoc. J..

[28] Abdelouahab N., Heard C. (2008). Effect of the major glycosides of *Harpagophytum procumbens* (Devil’s Claw) on epidermal cyclooxygenase-2 (*COX-2*). Vitro. J. Nat. Prod..

[29] Gyurkovska V., Alipieva K., Maciuk A., Dimitrova P., Ivanovska N., Haas C., Bley T., Georgiev M. (2011). Anti–inflammatory activity of Devil’s Claw in vitro systems and their active constituents. Food Chem..

[30] Jang M.-H., Lim S., Han S.-M., Park H.-J., Shin I., Kim J.-W., Kim N.-J., Lee J.-S., Kim K.-A., Kim C.-J. (2003). *Harpagophytum procumbens* suppresses lipopolysaccharide–stimulated expressions of cyclooxygenase-2 and inducible nitric oxide synthase in fibroblast cell line L929. J. Pharmacol. Sci..

[31] Fiebich B.L., Heinrich M., Hiller K.O., Kammerer N. (2001). Inhibition of TNF-α synthesis in LPS–stimulated primary human monocytes by *Harpagophytum* extract SteiHap 69. Phytomedicine.

[32] Inaba K., Murata K., Naruto S., Matsuda H. (2010). Inhibitory effects of Devil’s Claw (secondary root of *Harpagophytum procumbens*) extract and harpagoside on cytokine production in mouse macrophages. J. Nat. Med..

[33] Georgiev M.I., Ivanovska N., Alipieva K., Dimitrova P., Verpoorte R. (2013). Harpagoside: From Kalahari Desert to pharmacy shelf. Phytochemistry.

[34] Ichim M.C., Häser A., Nick P. (2020). Microscopic authentication of commercial herbal products in the globalized market: Potential and limitations. Front. Pharmacol..

[35] Grazina L., Amaral J.S., Mafra I. (2020). Botanical origin authentication of dietary supplements by DNA–based approaches. Compr. Rev. Food Sci. Food Saf..

[36] Raclariu A.C., Heinrich M., Ichim M.C., Boer H. (2018). Benefits and limitations of DNA barcoding and metabarcoding in herbal product authentication. Phytochem. Anal..

[37] Anantha Narayana D.B., Johnson S.T. (2019). DNA Barcoding in authentication of herbal raw materials, extracts and dietary supplements: A perspective. Plant Biotechnol. Rep..

[38] Grant L., McBean D.E., Fyfe L., Warnock A.M. (2007). A review of the biological and potential therapeutic actions of *Harpagophytum procumbens*. Phytother. Res..

[39] Little D.P. (2010). A Unified index of sequence quality and contig overlap for DNA barcoding. Bioinformatics.

[40] Hanner R. (2009). Proposed Standards for BARCODE Records in INSDC (BRIs).

[41] Thiers B. Index Herbariorum: A Global Directory of Public Herbaria and Associated Staff. http://sweetgum.nybg.org/science/ih/.

[42] Kool A., de Boer H.J., Krüger Å., Rydberg A., Abbad A., Björk L., Martin G. (2012). Molecular identification of commercialized medicinal plants in Southern Morocco. PLoS ONE.

[43] Little D.P. (2011). DNA Barcode sequence identification incorporating taxonomic hierarchy and within taxon variability. PLoS ONE.

[44] Thorner R.M., Remein Q.R. (1961). Principals and Procedures in the Evaluation of Screening for Disease.

[45] McNemar Q. (1947). Note on the sampling error of the difference between correlated proportions or percentages. Psychometrika.

[46] Nixon K.C., Wheeler Q.D. (1990). An amplification of the phylogenetic species concept. Cladistics.

[47] Baker D.A., Stevenson D.W., Little D.P. (2012). DNA barcode identification of black cohosh herbal dietary supplements. J. AOAC Int..

[48] Little D.P. (2014). Authentication of *Ginkgo biloba* herbal dietary supplements using DNA barcoding. Genome.

[49] Little D.P., Jeanson M.L. (2013). DNA barcode authentication of saw palmetto herbal dietary supplements. Sci. Rep..

[50] Busconi M., Foroni C., Corradi M., Bongiorni C., Cattapan F., Fogher C. (2003). DNA Extraction from olive oil and its use in the identification of the production cultivar. Food Chem..

[51] Fernandes T.J.R., Oliveira M.B.P.P., Mafra I. (2013). Tracing transgenic maize as affected by breadmaking process and raw material for the production of a traditional maize bread, broa. Food Chem..

[52] Gryson N., Messens K., Dewettinck K. (2008). PCR detection of soy ingredients in bread. Eur. Food Res. Technol..

[53] Hellebrand M., Nagy M., Morsel J.T. (1998). Determination of DNA traces in rapeseed oil. Z. Lebensm. Forsch. A.

[54] Meyer R. (1999). Development and application of DNA analytical methods for the detection of GMOs in food. Food Control.

[55] Murray S.R., Butler R.C., Hardacre A.K., Timmerman–Vaughan G.M. (2007). Use of quantitative real-time PCR to estimate maize endogenous DNA degradation after cooking and extrusion or in food products. J. Agric. Food Chem..

[56] Oguchi T., Onishi M., Chikagawa Y., Kodama T., Suzuki E., Kasahara M., Akiyama H., Teshima R., Futo S., Hino A. (2009). Investigation of residual DNAs in sugar from sugar beet (*Beta vulgaris,* L.). Food Hyg. Saf. Sci..

[57] Staats M., Cuenca A., Richardson J.E., Vrielink–van Ginkel R., Petersen G., Seberg O., Bakker F.T. (2011). DNA damage in plant herbarium tissue. PLoS ONE.

[58] Tilley M. (2004). PCR amplification of wheat sequences from DNA extracted during milling and baking. Cereal Chem..

[59] Bryan G.J., Dixon A., Gale M.D., Wiseman G. (1998). A PCR–based method for the detection of hexaploid bread wheat adulteration of durum wheat and pasta. J. Cereal Sci..

[60] Hupfer C., Hotzel H., Sachse K., Engel K.-H. (1998). Detection of the genetic modification in heat–treated products of *Bt* maize by polymerase chain reaction. Z. Lebensm. Forsch. A.

[61] Straub J.A., Hertel C., Hammes W.P. (1999). Limits of a PCR–based detection method for genetically modified soya beans in wheat bread production. Z. Lebensm. Forsch. A.

[62] Bauer T., Weller P., Hammes W.P., Hertel C. (2003). The effect of processing parameters on DNA degradation in food. Eur. Food Res. Technol..

[63] Duggan P.S., Chambers P.A., Heritage J., Forbes J.M. (2003). Fate of genetically modified maize DNA in the oral cavity and rumen of sheep. Br. J. Nutr..

[64] Sandberg M., Lundberg L., Ferm M., Malmheden Yman I. (2003). Real time PCR for the detection and discrimination of cereal contamination in gluten free foods. Eur. Food Res. Technol..

[65] Chen Y., Wang Y., Ge Y., Xu B. (2005). Degradation of endogenous and exogenous genes of roundup–ready soybean during food processing. J. Agric. Food Chem..

[66] Bergerová E., Godalova Z., Siekel P. (2011). Combined effects of temperature, pressure and low pH on the amplification of DNA of plant derived foods. Czech J. Food Sci..

[67] Bergerová E., Hrncirova Z., Stankovska M., Lopasovska M., Siekel P. (2010). Effect of thermal treatment on the amplification and quantification of transgenic and non–transgenic soybean and maize DNA. Food Anal. Methods.

[68] Costa J., Mafra I., Amaral J.S., Oliveira M.B.P.P. (2010). Monitoring genetically modified soybean along the industrial soybean oil extraction and refining processes by polymerase chain reaction techniques. Food Res. Int..

[69] Särkinen T., Staats M., Richardson J.E., Cowan R.S., Bakker F.T. (2012). How to open the treasure chest? Optimising DNA extraction from herbarium specimens. PLoS ONE.

[70] Lu Z., Rubinsky M., Babajanian S., Zhang Y., Chang P., Swanson G. (2018). Visualization of DNA in highly processed botanical materials. Food Chem..

[71] Boiteux L.S., Fonseca M.E.N., Simon P.W. (1999). Effects of plant tissue and DNA purification method on randomly amplified polymorphic DNA-based genetic fingerprinting analysis in carrot. J. Am. Soc. Hortic. Sci..

[72] Bowman M.J., Simon P.W. (2013). Quantification of the relative abundance of plastome to nuclear genome in leaf and root tissues of carrot (*Daucus carota*, L.) using quantitative PCR. Plant. Mol. Biol. Rep..

[73] Schmidt A.H. (2005). Validation of a fast–HPLC method for the separation of iridoid glycosides to distinguish between the *Harpagophytum* species. J. Liq. Chromatogr. Relat. Technol..

[74] Mann H.B., Whitney D.R. (1947). On a Test of whether one of two random variables is stochastically larger than the other. Ann. Math. Stat..

[75] Ihlenfeldt H.-D., Kadereit J.W. (2004). Pedaliaceae. The Families and Genera of Vascular Plants VII: Flowering Plants; Dicotyledons: Lamiales (except Acanthaceae including Avicenniaceae).

[76] Gormley I.C., Bedigian D., Olmstead R.G. (2015). Phylogeny of Pedaliaceae and Martyniaceae and the placement of *Trapella* in Plantaginaceae s. l. Syst. Bot..

[77] Chen S., Yao H., Han J., Liu C., Song J., Shi L., Zhu Y., Ma X., Gao T., Pang X. (2010). Validation of the ITS2 region as a novel DNA barcode for identifying medicinal plant species. PLoS ONE.

[78] Sang T., Crawford D.J., Stuessy T.F. (1997). Chloroplast DNA phylogeny, reticulate evolution, and biogeography of *Paeonia* (Paeoniaceae). Am. J. Bot..

[79] Lledo M.D., Crespo M.B., Cameron K.M., Fay M.F., Chase M.W. (1998). Systematics of Plumbaginaceae based upon cladistic analysis of *rbcL* sequence data. Syst. Bot..

[80] Levin R.A., Wagner W.L., Hoch P.C., Nepokroeff M., Pires J.C., Zimmer E.A., Sytsma K.J. (2003). Family–level relationships of Onagraceae based on chloroplast *rbcL* and *ndhF* data. Am. J. Bot..

[81] Fazekas A.J., Burgess K.S., Kesanakurti P.R., Graham S.W., Newmaster S.G., Husband B.C., Percy D.M., Hajibabaei M., Barrett S.C.H. (2008). Multiple multilocus DNA barcodes from the plastid genome discriminate plant species equally well. PLoS ONE.

